# Relationship between impaired glucose metabolism and bone mineral density in patients with cystic fibrosis

**DOI:** 10.1007/s00431-025-06732-2

**Published:** 2026-01-16

**Authors:** Mert Uçar, Hande Turan, Azer Kılıç Başkan, İlayda Altun, Gökçe Velioğlu Haşlak, Hasan Karakaş, Zeynep Taşkın, Dilek Bingöl Aydın, Abdurrahman Zarif Güney, Ömer Faruk Beşer, Ayşe Ayzıt Kılınç Sakallı, Olcay Evliyaoğlu, Elvan Bayramoğlu

**Affiliations:** 1 Department of Pediatric Endocrinology, Cerrahpasa Medical School, Istanbul University-Cerrahpasa, Koca Mustafapasa Cd. No: 53, Cerrahpasa Tıp Fakultesi Fatih Yerleşkesi, Istanbul, 34098 Fatih Türkiye; 2 Department of Pediatric Pulmonology, Cerrahpasa Medical School, Istanbul University-Cerrahpasa, Istanbul, Türkiye; 3 Department of Pediatrics, Cerrahpasa Medical School, Istanbul University- Cerrahpasa, Istanbul, Türkiye; 4 Department of Pediatric Gastroenterology, Hepatology and Nutrition, Cerrahpasa Medical School, Istanbul University- Cerrahpasa, Istanbul, Türkiye

**Keywords:** Cystic fibrosis-related diabetes, Cystic fibrosis-related bone disease, Bone mineral density, Osteoporosis

## Abstract

Cystic fibrosis (CF) is a chronic genetic disorder characterized by pancreatic insufficiency and lung disease. Advancements in highly effective modulator therapies (HEMTs) have improved life expectancy, shifting the focus to endocrine comorbidities, such as CF-related diabetes (CFRD) and bone disease (CFRBD). Therefore, current guidelines recommend routine screening for diabetes and osteoporosis in people with cystic fibrosis (PwCF) starting from age of 10 years. Increased risk of osteoporosis has been shown in type 1 and type 2 diabetes; however, there are limited studies evaluating the impact of glucose metabolism disorders on osteoporosis in children with cystic fibrosis. Therefore, this study investigates the impact of glucose metabolism disorders on PwCF. This cross-sectional retrospective study included 81 PwCF, aged between 10 and 21 years, who were screened routinely for diabetes and bone metabolism between 2019 and 2024. Data on demographics, *CFTR* variants, glucose metabolism, and biochemical bone parameters, including calcium, phosphorus, ALP, PTH, vitamin D levels with bone mineral density (BMD) of L1-L4 lumber spine were analyzed. Cases were categorized as normal, indeterminate, impaired glucose tolerance (IGT), or CFRD based on OGTT. Statistical analyses were conducted to determine factors affecting BMD, including pairwise comparison and multivariate regression analysis. Of the 81 cases, 55 (67.9%) had normal glucose tolerance, 9 (11.1%) had indeterminate (INDET), 9 (11.1%) had impaired glucose tolerance (IGT), and 8 (9.9%) had CFRD. IGT and CFRD cases demonstrated significantly lower body mass index (BMI), lung function, and BMD z-score than the normal group (*p* < 0.05). HbA1c had the negative association with BMD z-score (*β* = -0.36 per %1 increase in HbA1c, *p* < 0.001), while elevated BMI levels had positive relation (*β* = 0.28 per 1 kg/m^2^ increase in BMI, *p* = 0.009*)*. HEMT showed no significant impact on glucose or bone metabolism, likely due to short treatment durations.

*Conclusions*: Impaired glucose metabolism has a significant association with BMD in PwCF. Integrated monitoring of glucose and bone metabolism, along with a multidisciplinary approach is essential to optimize outcomes and reduce complications.

**Table:** 

**What is Known**:
• *Cystic fibrosis-related diabetes (CFRD) is the most common non-pulmonary comorbidity in CF*. • *Impaired glucose metabolism has been associated with reduced bone mineral density and increased fracture risk.*
**What is New**:
• *This study demonstrates that even early glucose metabolism disorders are associated with reduced bone mineral density in CF patients*. • *Higher HbA1c levels were found to be associated with lower bone mineral density, highlighting the relationship between hyperglycemia and bone health in CF*.

**Supplementary Information:**

The online version contains supplementary material available at 10.1007/s00431-025-06732-2.

## Introduction

Cystic fibrosis (CF) is a genetic disorder characterized by progressive lung disease and exocrine pancreatic insufficiency caused by variants in the cystic fibrosis transmembrane conductance regulator (*CFTR*) gene. Advancements in highly effective CFTR modulator therapies (HEMT) and supportive care have increased life expectancy [1]. Therefore, endocrine comorbidities, including cystic fibrosis-related diabetes (CFRD) and bone disease (CFRBD), have become increasingly important [1, 2].

CFRD is a distinct type of diabetes characterized by insulin deficiency and resistance features. The prevalence of CFRD increases with age [3]. CFRD is the most common extrapulmonary comorbidity, and it has been associated with accelerated pulmonary function decline [4, 5]. The mortality rate in cases with CFRD is higher compared to individuals with CF alone [6]. The pathophysiology of CFRD is intricate. The prevailing hypothesis suggests that collateral damage to islet cells, resulting from exocrine tissue injury, reduced ß-cell progenitor proliferation, and decreased survival are the causes of insulin deficiency [7, 8]. However, various other factors including genetic predisposition, inflammation, and malnutrition are also thought to contribute to the condition. The frequency of islet antibodies is similar to that in the general population, suggesting that autoimmunity does not play a role in the development of CFRD [9]. The diagnostic criteria for CFRD are the same as those used to diagnose other forms of diabetes, but low or normal HbA1c levels do not exclude the diagnosis. Given the potential for CFRD to be clinically insidious, routine screening is recommended for patients with cystic fibrosis. The standard oral glucose tolerance test (OGTT) is recommended for annual screening, starting at age 10 [10].

Another common long-term complication of CF is the cystic fibrosis-related bone disease (CFRDB), which can lead to severe morbidity [11]. The etiology of bone disease in patients with CF is also multifactorial, including the severity of genetic variants, inflammation, malabsorption, steroid therapy, and decreased physical activity [12]. CFRBD typically begins in childhood and progresses with age and disease severity. Early detection and management are essential to reduce fractures; therefore, the guidelines recommend routine bone mineral density (BMD) screening in all PwCF starting at 8–10 years of age [13].

The anabolic effect of insulin on bone and the reduced BMD in individuals with impaired glucose metabolism are well recognized. Studies have shown an increased risk of osteoporosis in individuals with insulin deficiency (type 1 diabetes, T1D) or insulin resistance (type 2 diabetes, T2D) [14]. The mechanism underlying the relationship between insulin deficiency and bone metabolism remains unclear. In this study, we aimed to elucidate the relationship of glucose metabolism disorders on BMD in cystic fibrosis cases.

## Methods

The present study is a retrospective cross-sectional analysis of 81 cystic fibrosis cases, aged between 10 and 21 years, who were followed at a single tertiary pediatric care center and screened for diabetes and bone disease between 2019 and 2024. The study included cases in which both OGTT and BMD screenings were performed with an interval of less than three months between two assessments. For cases with repeated screenings, only the most recent set of results that met these criteria was included in the analysis. Concurrent chronic disorders, acute exacerbations, glucocorticoid usage during the screening, and the absence of screening tests in the previous 5 years were considered exclusion criteria.

Ethical approval was granted by the Istanbul University- Cerrahpaşa Clinical Research Ethical Committee (Approval No: 2024–1157976). Written informed consent was obtained from legal guardians for participants under 18 years of age, and directly from participants aged 18 years and above. The study was conducted by the ethical principles outlined in the Declaration of Helsinki.

Demographic data, anthropometric measurements, *CFTR* variant analysis, glucose metabolism (blood glucose levels (BGL) and insulin levels during oral glucose tolerance test, fasting C-peptide, and HbA1c), bone metabolism (calcium (Ca), phosphorus (P), parathormone (PTH), 25-hydroxyvitamin D_3_ (25-OHD_3_) and BMD z-score), and spirometry (predicted forced expiratory volume (FEV1), forced vital capacity (FVC) and FEV1/FVC ratio) parameters were obtained retrospectively. Height, weight, and body mass index (BMI) standard deviation scores (SDS) are determined according to national growth references [15]. BMD was measured using the dual energy X-ray absorptiometry (DXA) method with Hologic Horizon W (Hologic, Marlborough, MA) and the mean lumber spine (L1-L4) BMD values were determined. The BMD z-scores were calculated using the manufacturer’s reference ranges adjusted for age, sex and height.

Oral glucose tolerance test results were assessed based on the International Society for Pediatric and Adolescent Diabetes (ISPAD) Clinical Practice Consensus Guidelines 2022 [16]. Cases were evaluated based on fasting plasma glucose (FPG), 1-h postprandial blood glucose levels (mid-OGTT), and 2-h postprandial BGL. Since all cases included in the study had FPG < 126 m/dl, the cases were divided into four groups: Cases with mid-OGTT < 200 mg/dl (11.1 mmol/l) and 2-h BGL < 140 mg/dl (7.8 mmol/l) were classified as normal, mid-OGTT ≥ 200 mg/dl (11.1 mmol/l) and 2-h BGL < 140 mg/dl (7.8 mmol/l) as indeterminate (INDET), 2-h BGL between 140–200 mg/dl (7.8–11 mmol(l) as impaired glucose tolerance (IGT) and 2 h BGL ≥ 200 mg/dl (11.1 mmol/l) as CFRD. The INDET, IGT, and CFRD groups were then defined as the “dysglycemic” to compare normal and impaired glucose metabolism. The differences in CFTR variant characteristics and HEMT usage between these groups were also analyzed.

### Statistical analysis

Statistical analysis was performed using SPSS version 29.0 (IBM Inc., Chicago, IL, USA) and GraphPad Prism version 10 (GraphPad Software, Inc., San Diego, CA, USA). *p*-values less than 0.05 were accepted as statistically significant. The Kolmogorov–Smirnov test was used to assess the normality. Descriptive parametric data were expressed as mean, and standard deviation values and non-parametric data were expressed as median and interquartile range. Student t-test was used to compare the normal and dysglycemic groups. Pearson chi-squared test (with Bonferroni correction) was used to analyze categorical data, such as CFTR variant analysis and HEMT usage The ANOVA test was used to compare the groups according to OGTT results. The post hoc Bonferroni test was used to analyze pairwise comparisons among these groups. Comparisons between groups were visualized using box plots. Pearson chi-squared test (with Bonferroni correction) was used to analyze categorical data, such as CFTR variant analysis and HEMT usage. Correlation analysis was assessed using Pearson’s correlation between anthropometrics, bone, and glucose metabolism parameters. Backward stepwise regression analysis was performed using variables correlated with the BMD z-score to identify the most fitting model.

## Results

The study included 81 cystic fibrosis cases, 36 females (43.2%) and 45 males (56.8%). The demographic and clinical data categorized by sex are summarized in Table 1. Females had significantly higher height SD values (*p* = 0.04), and males had higher 25-OHD_3_ levels (*p* = 0.03). No significant differences were observed based in other parameters based on sex.

**Table 1:** The demographic and clinical characteristics of cases stratified by gender

	Total (*n* = 81)	Female (*n* = 35, 43.2%)	Male (*n* = 46, 56.8%)	*p* value
Age (years)	14.8 ± 4	14.5 ± 4	15 ± 4	0.63
Weight SD	−0.9 ± 1.2	−0.62 ± 1.1	−1.11 ± 1.28	0.08
Height SD	−0.4 ± 1.1	−0.07 ± 0.84	−0.58 ± 1.27	**0.04**
BMI SD	−1 ± 1.3	−1 ± 1.5	−1.1 ± 1.32	0.84
FEV1 (predicted %)	80.5 ± 24	81 ± 22.8	80 ± 25	0.85
FVC (predicted %)	88 ± 20.7	88.5 ± 19	87.4 ± 22	0.80
FPG (mg/dl)	76.7 ± 11.1	76.8 ± 12.5	76.8 ± 10.1	0.99
Insulin (μU/mL)	5.8 ± 4.6	6.04 ± 4.41	5.67 ± 4.76	0.72
C-peptide (ng/ml)	2.13 ± 1.4	2.32 ± 1.6	1.97 ± 1.28	0.32
OGTT 2 h BGL	120 ± 41.7	127 ± 45.4	115.8 ± 38.5	0.24
HbA1c %	5.5 ± 0.5	5.5 ± 0.5	5.5 ± 0.6	0.96
Ca (mg/dl)	9.44 ± 0.4	9.37 ± 0.41	9.49 ± 0.38	0.18
P (mg/dl)	4.33 ± 0.6	4.33 ± 0.54	4.34 ± 0.6	0.96
PTH (pg/ml)	34.6 ± 16	34.8 ± 15.8	34.3 ± 16.5	0.93
25-OHD_3_ (ng/dl)	27.7 ± 14	23.5 ± 12.8	30.9 ± 14.1	**0.02**
BMD z-score	−1.25 ± 1.1	−1 ± 1	−1.4 ± 1.2	0.09

*BGL*: Blood glucose levels; *BMD*: Bone mineral density; *BMI*: Body mass index; *FEV1*: Forced expiratory volume; *FPG*: Fasting plasma glucose; *FVC*: Forced vital capacity; *OGTT*: Oral glucose tolerance test; *SD*: Standard deviation

*CFTR gene* analysis was performed in all cases, and variants were identified in 74 cases (91.1%). Among these, 23 (31.1%) had heterozygous, 14 (18.9%) had compound heterozygous, and 37 (50%) had homozygous variants. The most common variants were *F508del* (29.6%) and *E92K* (10.8%). Highly effective modulator therapy was administered in 12 cases (14.8%), with a mean duration of 1.3 ± 0.9 years. No significant difference was found in any variables when comparing the HEMT and non-HEMT groups.

**Fig. 1:**
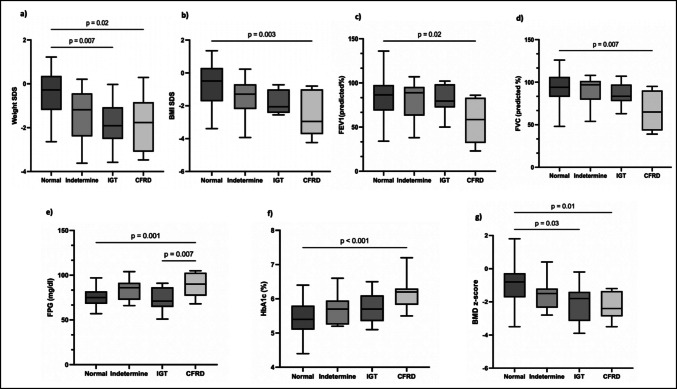
Comparative analysis of clinical and metabolic parameters among cases classified into normal, indeterminate, impaired glucose tolerance (IGT), and cystic fibrosis-related diabetes (CFRD) groups based on oral glucose tolerance test results. (**a**) Weight standard deviation scores (SDS), (**b**) body mass index (BMI) SDS, (**c**) predicted forced expiratory volume in 1 s (FEV1, %), (**d**) predicted forced vital capacity (FVC, %), (**e**) fasting plasma glucose (FPG, mg/dl), (**f**) HbA1c (%), and (**g**) BDM z-scores. Statistically significant differences (*p*-values < 0.05) are indicated between groups. CFRD cases demonstrate significantly lower weight SDS, BMI SDS, FEV1, FVC, and BMD z-scores, beside higher HbA1c and FPG levels

The cases were classified into four groups based on the OGTT results: 55 (67.9%) had normal glucose tolerance (NGT), 9 (11.1%) had indeterminate, 9 (11.1%) had IGT, and 8 (9.9%) had CFRD. The demographic and clinical characteristics of these groups are summarized in Table 2. Significant differences were observed in weight SD (*p* = 0.001), BMI SD (*p* = 0.004), FVC (*p* = 0.012), FPG (*p* = 0.001), HbA1c (*p* = 0.02), OGTT 2-h BGL (*p* = 0.001) and BMD z-score (*p* = 0.001). Statistically significant differences from pairwise comparisons are illustrated in Fig. 1. Weight, BMI, FEV1, FVC, and BMD z-scores were lower, while HbA1c and FPG levels were higher in CFRD cases than in the NGT group.

**Table 2:** The demographic and clinical characteristic of subgroups based on oral glucose tolerance test results

	Normal (*n* = 55, 67.9%)	INDET (*n* = 9, 11.1%)	IGT (*n* = 9, 11.1%)	CFRD (*n* = 8, 9.9%)	*p* value
Age (years)	14 ± 4	13.3 ± 3	15.9 ± 3.7	17.2 ± 4.2	0.125
Weight SD	−0.5 ± 1.1	−1.36 ± 1.3	−1.9 ± 1.1	−1.8 ± 1.3	**0.001**
Height SD	−0.2 ± 1.1	−0.9 ± 1.1	−0.9 ± 1.5	−0.5 ± 1.2	0.160
BMI SD	−0.7 ± 1.3	−1.5 ± 1.2	−1.8 ± 0.7	−2 ± 1.7	**0.004**
FEV1 (%predicted)	84 ± 23.4	81 ± 22.2	81.5 ± 17	57 ± 26	**0.030**
FVC (%predicted)	91 ± 20.2	89 ± 16.9	86 ± 13.8	66 ± 22.9	**0.012**
FPG (mg/dl)	75 ± 8.7	84 ± 12	73 ± 13.2	89 ± 13.4	**0.001**
Insulin (μU/mL)	6.5 ± 4.9	4.5 ± 4	4.6 ± 3.8	3.7 ± 2.5	0.235
C-peptide (ng/ml)	2.3 ± 1.4	1.8 ± 1.5	1.5 ± 0.9	2 ± 1.8	0.528
HbA1c %	5.4 ± 0.5	5.7 ± 0.5	5.7 ± 0.5	6.1 ± 0.5	**0.001**
Ca (mg/dl)	9.5 ± 0.4	9.55 ± 0.34	9.31 ± 0.44	9.19 ± 0.53	0.126
P (mg/dl)	4.3 ± 0.6	4.5 ± 0.4	4.2 ± 0.5	4.53 ± 0.8	0.344
PTH (pg/ml)	34.7 ± 17.6	33 ± 11.2	38.7 ± 20.8	31.6 ± 12.4	0.685
25-OHD_3_ (ng/dl)	28.7 ± 14.5	26 ± 11.1	27.2 ± 17.5	23.1 ± 7.4	0.806
BMD z-score	−0.9 ± 1.1	−1.6 ± 0.9	−2 ± 1.1	−2.2 ± 0.8	**0.001**

*BGL*: Blood glucose levels; *BMD*: Bone mineral density; *BMI*: Body mass index; *CFRD*: Cystic fibrosis related diabetes; *FEV1*: Forced expiratory volume; *FPG*: Fasting plasma glucose; *FVC*: Forced vital capacity; *IGT*: Impaired glucose tolerance; *INDET*: Indeterminate; *OGTT*: Oral glucose tolerance test

The demographic and clinical data of the dysglycemic group are summarized in Supplementary Table 1. Age (*p* = 0.02), HbA1c (*p* = 0.001), and FPG (*p* = 0.006) values were higher in the dysglycemic group, whereas weight SD (*p* = 0.001), BMI SD (*p* = 0.001), FVC (*p* = 0.03), fasting insulin levels (*p* = 0.04), and L1-L4 lumbar spine BMD z-score (*p* = 0.001) were higher in the NGT group. There were no differences in sex (*p* = 0.400), CFTR variant analysis (*p* = 0.600), F508del carriage (*p* = 0.187), or HEMT usage (*p* = 0.884) between the NGT and dysglycemic groups.

Oral glucose tolerance test results of 8 cases were available before and after HEMT. Among these, one transitioned from the INDET group to the IGT group, and CFRD was developed in a case from the IGT group. The remaining six cases remained in the NGT group both before and after treatment. No statistically significant differences were observed in HbA1c levels (*p* = 0.941) or BMD Z-scores (*p* = 0.641) of the cases with HEMT before and after treatment.

**Fig. 2:**
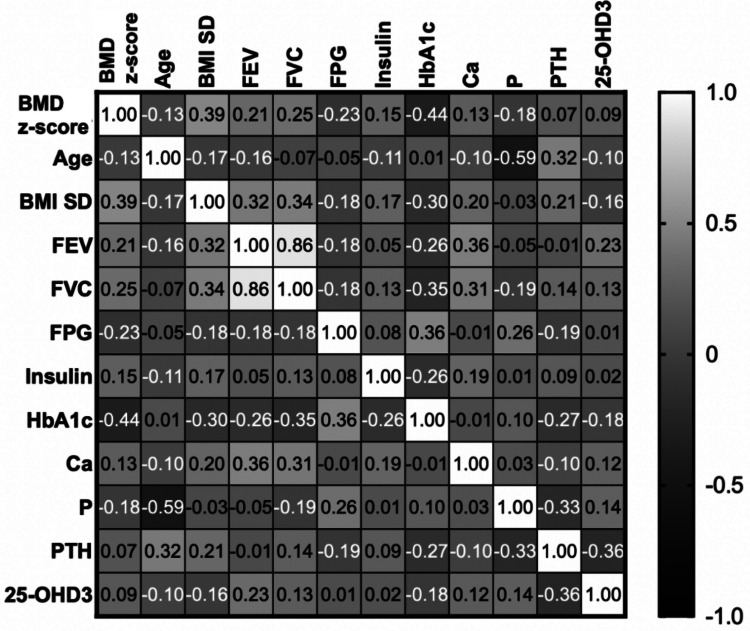
Correlation matrix displaying relationships among (BMD z-score, anthropometric parameters, pulmonary function (FEV, FVC), glucose metabolism (FPG, insulin, HbA1c), and bone metabolism parameters (Ca, P, PTH, 25-OHD3) in cystic fibrosis patients. Positive correlations are shown in lighter shades, while negative correlations are represented by darker shades. BMD z-score exhibits a positive correlation with BMI SD (R = 0.39) and FEV (R = 0.21) but a negative correlation with HbA1c (R = −0.44)

Correlation analysis was conducted to determine the relationships among anthropometric measurements, glucose, and bone metabolism parameters, depicted in Fig. 2. The lumbar spine BMD z-score demonstrated a weak positive correlation with BMI SD and a moderate negative correlation with HbA1c. The results of backward stepwise regression modeling are summarized in Table 3. Although all models were statistically significant, HbA1c, BMI SD, and phosphate levels emerged as the most relevant variables influencing the BMD z-score. Notably, HbA1c had a significantly negative association on the BMD z-score, while BMI SD was associated with a positive relationship.

**Table 3:** Multivariate linear regression models of BMD z-score and explanatory variables

Constant	Explanatory Variables	Adjusted R^2^	Standardized β	*p*-value
Model 1				< 0.001**
BMD z-score	HbA1c	0.226	−0.34	*0.004*
	BMI SD		0.26	*0.018*
	FEV1		0.03	0.882
	FVC		−0.01	0.982
	FPG		−0.07	0.510
Model 2				< 0.001*
BMD z-score	HbA1c	0.246	−0.34	*0.003*
	BMI SD		0.26	*0.017*
	FEV1		0.02	0.812
	FPG		−0.10	0.506
Model 3				< 0.001**
BMD z-score	HbA1c		−0.36	< *0.001*
	BMI SD	0.251	0.28	*0.009*

*BMD*: Bone mineral density; *BMI*: Body mass index; *FEV1*: Forced expiratory volume in 1 s; *FVC*: Forced vital capacity

## Discussion

This study demonstrated that HbA1c and BMI SD were significantly associated with BMD in patients with cystic fibrosis. Elevated HbA1c levels have a negative association with the BMD z-score, while BMI SD has a positive relation.

Glucose metabolism disorders have a negative impact on bone metabolism, as demonstrated in patients with both type 1 and type 2 diabetes mellitus [17]. Insulin promotes osteoblast function and bone tissue formation by enhancing DNA synthesis, osteocalcin production, and collagen formation. In addition to insulin deficiency, hyperglycemia directly inhibits the activity of osteoblast and osteocyte. Elevated glucose levels impair bone metabolism by reducing the synthesis of extracellular matrix components and the bone mineralization process [18].

Glucose metabolism impairment initially manifests as postprandial hyperglycemia due to the loss of the first phase of insulin secretion. This phase is followed by impaired glucose tolerance, and with further progression, diabetes eventually develops [19]. Therefore, even before the onset of CFRD, hyperglycemia may contribute to a decrease in BMD. Our study demonstrated that the NGT group had higher BMD than cases with impaired glucose metabolism. Additionally, no difference in BMD was observed among the groups with impaired glucose metabolism. These findings suggest that the impaired glucose metabolism, even at an early stage, may be associated with lower BMD in PwCF.

There are only a limited number of studies evaluating this relationship in cystic fibrosis. A study that included adult cystic fibrosis patients showed that bone turnover markers were decreased in CFRD and negatively associated with glucose levels [20]. In a study involving young cystic fibrosis patients, the IGT/CFRD group had a significantly lower z-score than the NGT group; however, the IGT and CFRD groups were not evaluated separately [21]. Our study demonstrated that increased mean glucose levels negatively impacted the BMD in cystic fibrosis patients.

Contrary to HbA1c, BMI SD has a positive effect on bone health. A recent study in patients with cystic fibrosis showed that cases with low BMD had similar height but lower weight and BMI z-score compared to the normal group [22]. It is noteworthy that weight and BMI SD values were lower in the IGT and CFRD groups compared to the normal group. In a meta-analysis evaluating the relationship between BMI and clinical findings in adult cystic fibrosis, it was found that the risk of CFRD increased in individuals with low BMI [23].

In our study, a weak correlation was observed between the BMD z-score and biochemical bone parameters, including calcium, phosphorus, ALP, PTH, and vitamin D levels. Additionally, no significant differences were observed in these parameters between the NGT group and the dysglycemic group. As demonstrated in the previously mentioned study, our study also found no direct relationship between calcium metabolism parameters and BMD in cystic fibrosis [21].

It is known that FEV1 and FVC decrease over time in the natural course of cystic fibrosis. However, studies have shown that the lung capacity of CFRD is worse than cases without diabetes. In our study, it was demonstrated that predicted FEV1 and FVC values of CFRD cases were significantly lower than the normal group. It should be noted that although it is known that indeterminate cases have an increased risk of progression to diabetes, the lung function and BMD of these patients were not different from the normal cases in our study.

Consistent with the national registry system and other studies, the most common allele identified in our study was *F508del*. However, the second most common allele was *E92K* rather than *N1303K* [24, 25]*.* It has been shown in the literature that the incidence of IGT and CFRD is higher in patients with a severe genotype, especially individuals with homozygous the *F508del* [6, 26, 27]. In this study, no difference was found among the dysglycemic and normal groups in the presence of *F508del* carriage and homozygous variant.

No differences were observed in HbA1c and BMD values before and after high-efficacy modulator therapy in our study; however, it should be noted that the number of PwCF receiving HEMT and the duration of therapies were limited. In the literature, findings regarding changes in glucose metabolism and bone density before and after treatment were controversial. Among two studies evaluating BMD after three months of HEMT, one demonstrated a beneficial effect of the treatment, while the other reported similar BMD levels before and after treatment [28, 29]. This study contributes additional information to the literature by assessing a comparable number of patients after a longer treatment duration. In different studies evaluating CF patients with HEMT, no significant differences were observed in glycemic status or CGM measurements before and after treatment [30, 31].

The retrospective nature of the study precludes a definitive establishment of causality; however, our findings provide evidence of a negative relationship between impaired glucose metabolism and bone health in PwCF. Other limitations of this study include a relatively small sample size and missing clinical data, such as corticosteroid use, Tanner staging, and dietary assessments. The use of HEMT in a small number of cases and for a relatively short period is a limitation in the valuation of treatment efficacy. Future research with larger cohorts and more extended follow-ups is needed to evaluate the long-term effects of HEMT.

## Conclusion

In conclusion, this study demonstrated the significant negative relationship between impaired glucose metabolism on bone health in cystic fibrosis, highlighting the necessity of early intervention to mitigate long-term skeletal complications. Optimized glycemic control and preventing malnutrition are essential for preserving BMD and improving patient outcomes. These findings underscore the importance of a multidisciplinary approach that integrates endocrinology, pulmonology, and nutrition, while also highlighting the need for CF-specific strategies to manage dysglycemia and its associated complications.

## Supplementary Information

Below is the link to the electronic supplementary material.

Supplementary file1 (DOCX 20 KB)

## Data Availability

The datasets generated and analyzed during the current study are available from corresponding author on request.

## Abbreviations

BGL: Blood glucose level
BMD: Bone mineral density
BMI: Body mass index
CFRBD: Cystic fibrosis-related bone disease
CFRD: Cystic fibrosis-related diabetes
CFTR: Cystic fibrosis transmembrane conductance regulator
FEV: Forced expiratory volume
FPG: Fasting plasma glucose
FVC: Forced vital capacity
HEMT: Highly effective CFTR modulator therapy
IGT: Impaired glucose tolerance
INDET: Indeterminate
OGTT: Oral glucose tolerance test
PwCF: People with cystic fibrosis
T1D: Type 1 diabetes
T2D: Type 2 diabetes
