# Supplementary material for: Relationship between impaired glucose metabolism and bone mineral density in patients with cystic fibrosis
Source: Eur J Pediatr. 2026 Jan 16;185(2):84. doi: 10.1007/s00431-025-06732-2 (PMC12811283; doi:10.1007/s00431-025-06732-2)
Supplement: Supplementary file 1 — Supplementary file1 (DOCX 20 KB) [file 431_2025_6732_MOESM1_ESM.docx]

**Supplementary Table 1.** The demographic and clinical charactericstics of normal and dysglycemic cases.

|  | **Total**  **(n=81)** | **Normal**  **(n=55, 67.9%)** | **Dysglycemic**  **(n=26, 32.1%)** | **p value** |
| --- | --- | --- | --- | --- |
| **Age (years)** | 14.8 ± 4 | 14 ± 4 | 16.2 ± 3.6 | **0.020** |
| **Weight SD** | -0.9 ± 1.2 | -0.5 ± 1.1 | -1.6 ± 1.2 | **0.001** |
| **Height SD** | -0.4 ± 1.1 | -0.2 ± 1.1 | -0.75 ± 1.24 | 0.052 |
| **BMI SD** | -1 ± 1.3 | -0.7 ± 1.3 | -1.8 ±1.24 | **0.001** |
| **FEV1 (predicted %)** | 80.5 ± 24 | 84 ± 23.4 | 73 ± 24 | 0.077 |
| **FVC (predicted %)** | 88 ± 20.7 | 91 ± 20.2 | 81 ± 20 | **0.030** |
| **FPG (mg/dl)** | 77 ± 11.1 | 75 ± 8.7 | 81.7 ± 14 | **0.006** |
| **Insulin (μU/mL)** | 5.8 ± 4.6 | 6.5 ± 4.9 | 4.3 ±3.7 | **0.040** |
| **C-peptide (ng/ml)** | 2.13 ± 1.4 | 2.3 ± 1.4 | 1.8 ± 1.5 | 0.200 |
| **HbA1c %** | 5.5 ± 0.5 | 5.4 ± 0.5 | 5.8 ± 0.5 | **0.001** |
| **Ca(mg/dl)** | 9.44 ± 0.4 | 9.48 ± 0.4 | 9.4 ± 0.4 | 0.249 |
| **P (mg/dl)** | 4.33 ± 0.6 | 4.3 ± 0.6 | 4.4 ± 0.6 | 0.517 |
| **PTH (pg/ml)** | 34.6 ± 16 | 34.7 ± 17.6 | 34.5 ± 14.4 | 0.976 |
| **25-OHD_3_ (ng/dl)** | 27.7 ± 14 | 28.7 ± 14.5 | 25.7 ± 13 | 0.373 |
| **DEXA z-score** | -1.21 ± 1.1 | -0.9 ± 1.1 | -1.9 ± 1 | **0.001** |

**BGL**: Blood glucose levels, **BMI**: Body mass index, **DEXA**: Dual energy x-ray absorptiometry, **FEV1**: Forced expiratory volume in 1 second, **FPG**: Fasting plasma glucose, **FVC**: Forced vital capacity, **OGTT**: Oral glucose tolerance test, **SD**: Standart deviation
